# Studying the impact of phycoerythrin on antioxidant and antimicrobial activity of the fresh rainbow trout fillets

**DOI:** 10.1038/s41598-024-52985-6

**Published:** 2024-01-30

**Authors:** Bahareh Nowruzi, Mahsa Ahmadi, Noureddine Bouaïcha, Amir Eghbal Khajerahimi, Seyed Amir Ali Anvar

**Affiliations:** 1 Department of Biotechnology, Science and Research Branch, Islamic Azad University, Tehran, Iran; 2 Department of Food Hygiene, Science and Research Branch, Islamic Azad University, Tehran, Iran; 3 Laboratory Ecology, Systematic and Evolution, UMR 8079, Universite Paris-Sud, CNRS, AgroParisTech, University Paris-Saclay, 91405 Orsay, France; 4 Department of Aquatic animal health and disease, science and research branch, Islamic Azad University, Tehran, Iran

**Keywords:** Biological techniques, Biotechnology, Microbiology, Plant sciences

## Abstract

Marine cyanobacteria present a significant potential source of new bioactive compounds with vast structural diversity and relevant antimicrobial and antioxidant activities. Phycobiliproteins (PBPs) like phycocyanin (PC), phycoerythrin (PE), and water-soluble cyanobacterial photosynthetic pigments, have exhibited strong pharmacological activities and been used as natural food additives. In this study, phycoerythrin (PE) isolated from a marine strain of cyanobacterium *Nostoc* sp. Ft salt, was applied for the first time as a natural antimicrobial as well as an antioxidant to increase the shelf life of fresh rainbow trout i.e., (*Oncorhynchus mykiss*) fillets. Fresh trout fillets were marinated in analytical grade PE (3.9 μg/mL) prepared in citric acid (4 mg/mL), and stored at 4 °C and 8 °C for 21 days. Microbiological analysis, antioxidant activity and organoleptic evaluation of both control and treated fish fillets were then statistically compared. The results demonstrated noticeable (P < 0.05) differences in the microbial counts, antioxidant activity, and organoleptic characteristic values between PE-treated and non-treated groups. In addition, we observed that treating fresh fish fillets with a PE solution leads to a significant increase in shelf life by at least 14 days. Consequently, PE could be an alternative to synthetic chemical additives since it does not contain the potentially dangerous residues of the synthetic chemical additives and is thus healthier to the consumers.

## Introduction

Fish is of great dietary importance because of its high protein, vitamins, and mineral content, and also its generally low saturated fats and calorie content^1,2^. With the intention of inhibiting microbial activity and extending the shelf life of food, the food industry is continually looking for practical and alternative and at the same time natural preservation technologies to store food due to the controversy over synthetic compounds^3^. It has been validated that using edible natural pigments^4^ extends the shelf life of fresh fish. It acts through making a protective barrier that shortens the interaction between the product and the atmosphere, decreases the operations of metabolic deterioration and delays microbial growing^5^. Additionally, the high level of polyunsaturated fatty acids in fish forces it to be extremely vulnerable to rancidity caused by free radicals in oxygen^6^. As a result, undesirable flavors and odors resulting from lipid oxidation decrease the quality of the fish, and therefore, shorten the shelf life of fish fillets during storage periods^7^. Sadly, a lot of additives that are used to control oxidative processes are ineffectual in lessening microbial growth. Also, potential antimicrobial substances may not maintain fish organoleptic parameters within reasonable limits^8^. Using edible natural pigments, exhibiting both antimicrobial and antioxidant activity, is an effective and safe means of minimizing or extending the shelf life of foods^9–12^. For exemple, Viera et al.^10^ revealed that the addition of cell extract of the cyanobacterial species *Arthrospira platensis* encapsulated in alginate microbeads to a 3D printed cookie, improved its thermal stability, its durability against oxygen and light when they are stored or baked.

Besides their traditional application as a human source of nutriment, some marine and freshwater cyanobacterial species have an immense potential as alternative sources of a various of bioactive compounds with promising broad pharmaceutical and food-related applications^13–16^. The phycobiliproteins (PBPs) like C-phycoerythrin (PE), C-allophycocyanin (APC), and phycocyanin (PC), are among the value-added bioactive metabolites produced by cyanobacteria, which have been used as food additives because of their proven antimicrobial and antioxidant potential^17^. The capacity of phycobiliproteins to function like free radical scavengers has been shown to center on their tetrapyrrols systems, contributing to their use in the cosmetics, pharmaceutical, and food industries^18,19^. Numerous accounts of analytical grade PBPs (purity ratio > 4.0) have shown that PBPs possess exceptional antioxidant, anti-inflammatory, and hepato-protective, activities^20–26^. In this research, the C-PE is purified and characterized from a marine cyanobacterial strain *Nostoc* sp. Ft salt (MG549320) isolated from *Caspian Sea* (Iran). The purified C-PE was tested in vitro for its antimicrobial, as well as its antioxidant activities, and it was evaluated for the first time to increase the shelf life of rainbow trout fillets.

## Materials and methods

### Chemicals

All protein and chemicals molecular weight markers applied in the present research were of analytical grade, acquired from the Hi-Media, Merck. Sigma, and Hi-Media, Merck manufacturers.

### Culture conditions of the cyanobacterial strain

Marine cyanobacterial strain *Nostoc* sp. Ftsalt (MG549320) were isolated from *Caspian Sea.* We gathered the samples a by a sterilized spatula from the sea surface up to 5 cm deep. Then we detached the surface debris, and transferred each sample aseptically to sterile petri dishes with BG11_0_ solid culture medium^27^. After 20 days, we singled out the isolated colonies, washed them with sterile deionized water and coveyed them to 1 mL of fresh BG11_0_ liquid medium. After 10–12 days in liquid culture medium, cells were plated on the sterile BG11_0_ solid medium again applying spread plate technique. We repeated the procedures until unicyanobacterial cultures were obtained. Thereafter, the strains were cultured in an Erlenmeyer flask holding 100 mL of liquid medium, with the pH modified to 7.2. Then we kept the cultures at 28 ± 2 °C and periodically shaked it under a photoperiod of 14:10 h (light:dark) cycle and illuminated them with ca. 50–55 µmol photons m^−2^ s^−1^. Strain observations were carried out with the help of an Olympus CX31RTS5 (Olympus, Japan) stereoscope provisioned with a QImaging GO-3 digital camera (Teledyne QIMAGING, Canada) and an Olympus BX43 provisioned with a Sc50 digital camera (Olympus, Japan)^28^. We placed fresh cultures and exsiccates at the ALBORZ Herbarium and the Cyanobacteria Culture Collection (CCC), in the Science and Research Branch, Islamic Azad University, Tehran.

### Extracting and purifying analytical grade of phycoerythrin

Nowruzi et al.^18^, conducted extracting and purifying phycoerythrin (PE). Briefly, In order to procure a pellet, after centrifuging at 4000 rpm, we removed PE from 500 mL of homogenized log phase culture which was 14 days old*.* We suspended the pellet in 100 mL of 20 mM acetate buffer (pH 5.1), and then for 4 days, we applied the continual freezing (− 20 °C) and thawing (room temperature) method until the cell biomass turned dark purple. We obtained a crude extract by detaching cell debris and then centrifugng at 5000 rpm for 10 min. We applied Afreen and Fatma method (2018)^29^ and purified the extract. Then we slowly added solid ammonium sulphate to the crude extract to achieve 65% saturation via stirring the extract continuously. After that, we let the resulting solution to stand in a cold room for 12 h, and centrifuged it at 4500×*g* for 10 min. We resuspended the pellets in a small volume of 50 m acetic acid–sodium acetate buffer (pH 7.1), and dialyzed it overnight. This way, we recovered the extract from the dialysis membrane and filtered it through 0.45 μm filter^30,31^.

### Determination of PE content and its purity ratio

We calculated the amounts of PE, PC and APC in the various extracts obtained by measuring the absorbance at 565, 620 and 650 nm using the following equations^32^. We calculated the purity of PE at each step by calculating the purity ratio (A555/A280) where absorbance at 280 nm and 555 nm showed the concentration of proteins and PE^32^. We noted the UV–visible spectrum over the wavelength range from 250 to 700 nm applying a U-2910 spectrophotometer (HITACHI Co., Japan) at room temperature^33^.

$${\text{PC }}\left( {\mu {\text{g mL}}^{{ - {\text{1}}}} } \right){\text{ }} = ~\frac{{\left( {{\text{OD~}}620{\text{nm}} - 0.7{\text{OD~}}650{\text{nm}}} \right)}}{{7.38}}~$$

$${\text{APC}}\,\left( {\mu {\text{g}}\,{\text{mL}}^{{{\text{ - 1}}}} } \right){\text{ = }}\left( {\frac{{{\text{OD}}\,{\text{650nm - 0}}{\text{.19OD620nm}}}}{{{\text{5}}{\text{.65}}}}} \right)$$

$${\text{PE}}\,\left( {{\mu \text{g}}\,{\text{mL}}^{{{\text{ - 1}}}} } \right){\text{ = }}\left( {\frac{{{\text{OD}}\,{\text{565nm}} - {\text{2}}{\text{.8}}\left[ {{\text{R}} - {\text{PC}}} \right] - {\text{1}}{\text{.34}}\left[ {{\text{APC}}} \right]}}{{{\text{12}}{\text{.7}}}}} \right)$$

### Effect of different preservatives and temperature on the stability of the analytical grade C-PE

We tested the stability of phycoerythrin by adding the following preservatives, calcium chloride, viz. sucrose, citric acid, and sodium chloride, kept at two temperatures (4 and 8 °C). To check the effectiveness of preservatives, calcium chloride (0.4 g), sucrose (0.4 g), sodium chloride (0.4 g), and citric acid (0.4 g) were added in 25 ml phycoerythrin solution. We dissolved 5 mg of freeze dried phycoerythrin in 25 ml of sodium phosphate buffer (0.1 M, pH 7.2) one at a time and kept it at 4 °C and 8 °C to study. The stability of PE was then measured by recording its absorption spectrum and determining the percentage of degration as described by ^34^.

### Estimatng the MIC (minimum inhibitory concentration( of the purified C-PE

Discover the suitable concentration for microbial analysis, the MIC of the purified analytical grade of the purified C-PE with and without adding citric acid (4 mg/mL) was decided based on the standard reference method^35^. The assays were repeated three times and each in duplicate.

### Preparation and treatment of fish fillets

Forty Fresh rainbow trout specimens (*Oncorhynchus mykiss*; average weight: 600 ± 650 g) were taken from the Caspian Sea and we kept them in ice while we were transporting them to the laboratory^36^. A total of 60 fillets were used for the study. First, we washed the fillets in tap water, and then divided them into two lots. The first lot contained 40 fillets, each with the size of 5 cm × 5 cm × 1 cm (width × length × thickness), and weighing 10 g, 20 fillets were used for microbiological analysis control and 20 other fillets a for sensory evaluation. The second lot contained 20 fillets each with the size of 8 cm × 8 cm × 1 cm (length × width × thickness) and weighing 25 g were used for detection of *Salmonella* sp. Each day of the experiment, a separate fillet of fish was used (0, 3, 7, 14 and 21 days) to evaluate the shelf life at different temperatures of storage (4 and 8 °C). According to the results of the MIC, 30 fillets were marinated in the analytical grade of the purified C-PE prepared in a citric acid solution (4 mg/mL) as preservative at an ultimate concentration of 3.9 μg/mL and the other fillets were used as control without C-PE. Then we packaged the treated fillets one at the time in polyethylene zipper bags and stored them at 4 °C and 8 °C in refrigerator for (0, 3, 7, 14, and 21 days). Extraction of each fish fillet was performed separately on the day of the experiment excepct for *salmonella* sp. where the fillets were used directly for testing. For extraction, sterile physiological serum (90 ml) was added to each fillet and homogenized in a Waring blender (Waring Products, Torrington, Conn., USA) for 1 min.

### Microbiological analysis

#### Total viable microorganisms count

We conducted total viable psychrophilic bacteria counts based on Mari and Antonini^37^ and demonstrated microbiological loads as number of colony-forming units (cfu) per gram.

#### Total psychrophilic bacteria count

We conducted total viable psychrophilic bacteria counts based on Raeisi et al.^38^. Briefly, We incubated plates of psychotropic bacteria for 10 days at 7 °C, and we counted them on Cetrimide Agar (CFC, Merck, Darmstadt, Germany) after incubating them for 48 h at 25 °C. Every count in this study is demonstrated as log colony-forming unit (cfu)/g and is carried out twice.

#### Staphylococcal coagulase-positive bacteria count

We conducted staphylococcal coagulase-positive bacteria counts based on Junior et al.^39^. Briefly, we laid out 0.1 ml samples of serial dilutions of fish tissue homogenates on the Baird Parker Agar surface at a temperature of 35–37 °C and incubated it for 24–48 h and then through the coagulase test, found out the suspected colonies.

#### Enumerating total coliforms

We used Most Probable Number (MPN) method to quantitatively estimatate total coliforms^40,41^. To do so, we needed needs at least 24 h, while for for gas negative tubes we needed an additional 24 h incubation of inoculated lauryl sulphate tryptose (LST) broth at 37 °C. Every gas positive tube needed to be converyed into brilliant green bile (BGB) broth and incubated for 24 h at the same temperature, so that it confirms to the presumptive coliform results aquired from LST broth.

#### Total fecal count (E.coli) test

To detect the presence or absence (positive or negative) of faecal coliform, a loopful of suspension from each of the previously incubated LST samples was transferred into a test tube with Brilliant Green Lactose Bile broth (BGLB- DIFCO ^™^ ) and (Escherichia coli broth (EC-DIFCO ^™^), and incubated at 35 °C for 48 h and 45.5 °C for 24 h, respectively. The positive test tubes were gently agitated and examined for gas formation or effervescence. The results were recorded as positive or negative for faecal coliform^41^ ^40^.

## Detecting *salmonella* spp.

We apllied Sanjee and Karim^41^ procedure to detect *Salmonella* spp.Briefly, An aliquot of 25 g of each fish fillet was dissolved in 225 mL of sterilized Lactose Broth (LB), blended, and incubated at 37 °C for 16–20 h. We restrictively enriched an aliquot of 1 mL from the incubated LB culture into the 10 mL sterilized Rappaport Vassiliadis Soya Broth and incubated it again for 24–48 h at 37 °C. Then we incubated, one loopful inoculum from the particular enrichment culture and streaked it onto the preincubated SS and XLD agar plate. Normal *Salmonella* spp. make pink colonies accompanied by or without black colonies on SS agar, and black centers on XLD agar. Then we spotted the suspected colonies by their biochemical, cultural, and morphological characteristics, through TSI (Triple Sugar Iron), Urease test, and Indole test^40^. The identified *Salmonella* spp. were serogrouped using a BD Difco™ Salmonella O Antisera in slide agglutination tests^41^.

### Antioxidant activity of the analytical grade of C-PE

The antioxidant activity of the analytical grade of C-PE was evaluated according to the 2, 2 diphenyl-1-dipicrylhydrazyl (DPPH) method described by Afreen and Fatma^29^ with little modifications*.* Briefly, we purified 710 μg/mL PE, mixed it with 1 mL of DPPH reagent. Then r incubated it for 30 min in the dark at room temperature, we measured the absorbance at 517 nm and used ascorbic acid (100 μg/mL) as positive control.

*Activity* (%) = *Ac* − *At*/*Ac* × 100

In which *At* is the absorbance of sample, and *Ac* is the absorbance of DPPH.

### Sensory evaluation

According to the results of microbial count, ten panelists assessed f the fish fillets quality marinated in the C-PE analytical solution without PE for control fillets on the days of 0th, 3rd, 7th, 14th. Their ensory characteristics like odor, color, texture, appearance, flavor, and overall acceptability was measured by using 5-point hedonic scale according to Fadıloglu and Çoban^42^. We heated the fillets of rainbow trout in an oven using an oven bag for 10 min at 180 °C just before serving them.

### Statistical study

We ascertained the effect of the categorical variables “additive”, “temperature”, and “storage time”, we also ascertained s their interactions, for each numeric parameter studied by SPSS 24, IBM Corporation Inc. The statistical difference reached the remarkable level of of 95% We carried out the Duncan t to evaluate the importance of the difference between mean values as we found a remarkable variation (*p* < 0.05) by the ANOVA test. Moreover, to compare the equality of means of independent variables (control and treated fillets + citric acid + PE), we applied Levene’s tests and t-tests at the notable 0.05 level. We performed three repeated assessments for every treatment where we secured the mean values ± standard error of mean.

### Statement

All methods were conducted congruent with relevant guidelines and regulations. Authors have not done do any experiments on humans and/or the use of human tissue samples. All experimental protocols and panelists involved in the study were accepted by ethics committee of Tehran medical sciences, Islamic Azad University, Tehran, Iran. Moreover evaluating panel sampled the rainbow trout (*Oncorhynchus mykiss*) fillets. The full name of the ethics committee that approved the study is Dr Fahimeh Nemati and Dr Sarvenaz Falsafi.

## Results

### Purification of PE and effects of temperature and stabilizers in its stability

**Figure 1:**
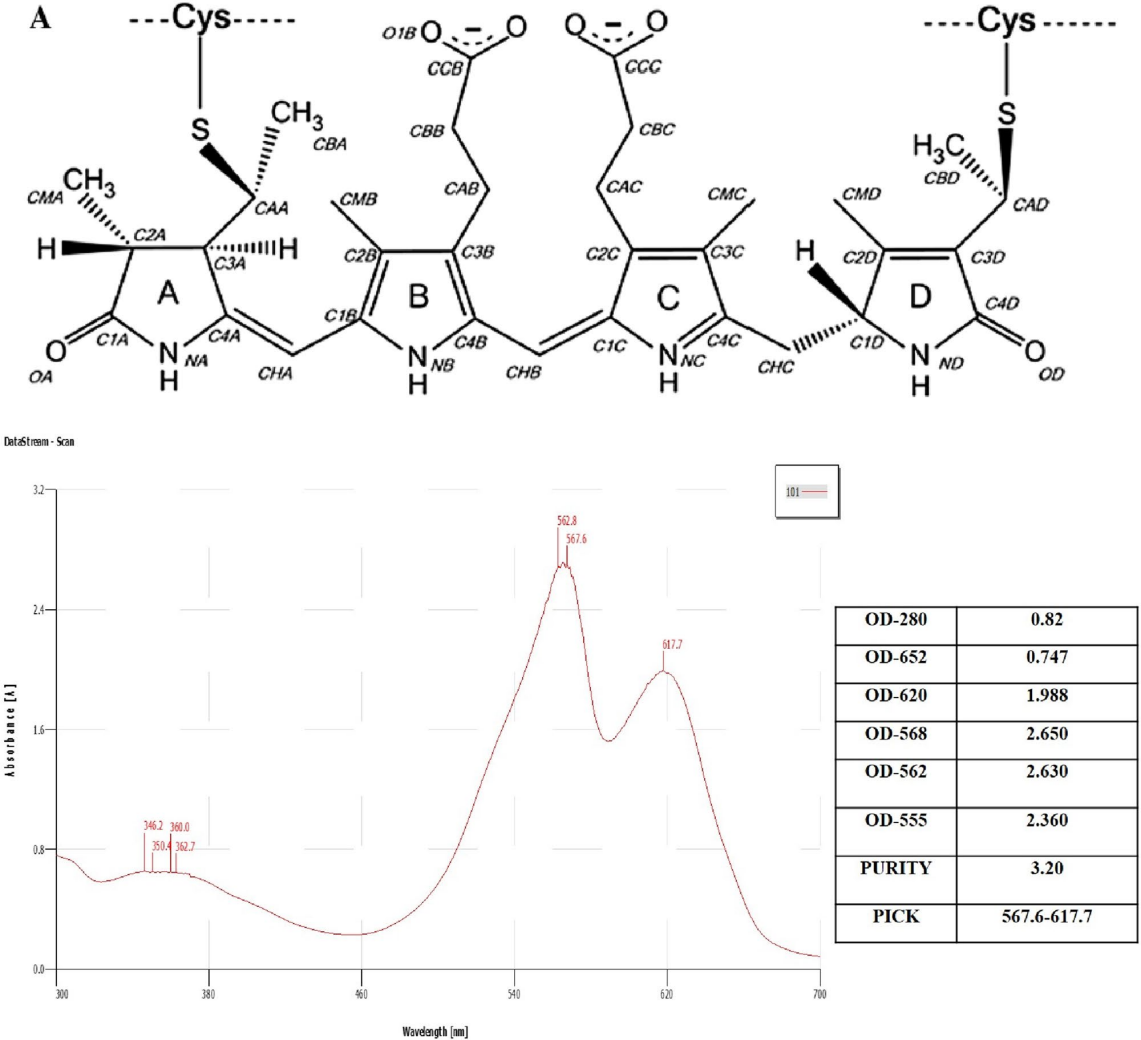
The general chemical structure of PE binds covalently to proteins via two thiol bonds to form PBPs and the UV–visible absorbance spectrum (below) of the purified analytical grade (C-PE) from *Nostoc* sp. Ft salt (MG549320).

The purity ratio of PE extracted from a marine cyanobacterial strain *Nostoc* sp. Ftsalt (MG549320), successevily with acetate buffer (pH-5.1), ammonium sulfate, and dialyze, was reported in Table 1 and Fig. 1. Conforming to the food grade requirement and after every purification step, the purity and concentration of PE were increased (Table 1). During the successive purification steps, purity ratio rose from 1.54 up to 4.88 (Table 1) and reached the analytical grade standard. Therefore, the purity from crude extract to purified PE grew by nearly four times,which suggested that the method is effective in securing high analytical purity of PE. The purity ratio of phycolibiproteins (PBPs) ibetween 0.7 to 2.9 is regarded to be food grade, the PBPs whose purity ratio ranges between 3.0 to 3.9 are the reagent grade more than 4.0 purity ratio is the analytical grade.

**Table 1:** Stepwise purification of PE from *Nostoc* sp. Ftsalt (MG549320).

Purification steps	Volume (ml)	PE (μg/mL)	PE Purity (OD555/OD280)
Crude extract in acetate buffer (pH 5.1)	100	60.04	1.54
Ammonium sulfate precipitation	10	510	4.61
Dialysis	10	565	4.88

**Figure 2:**
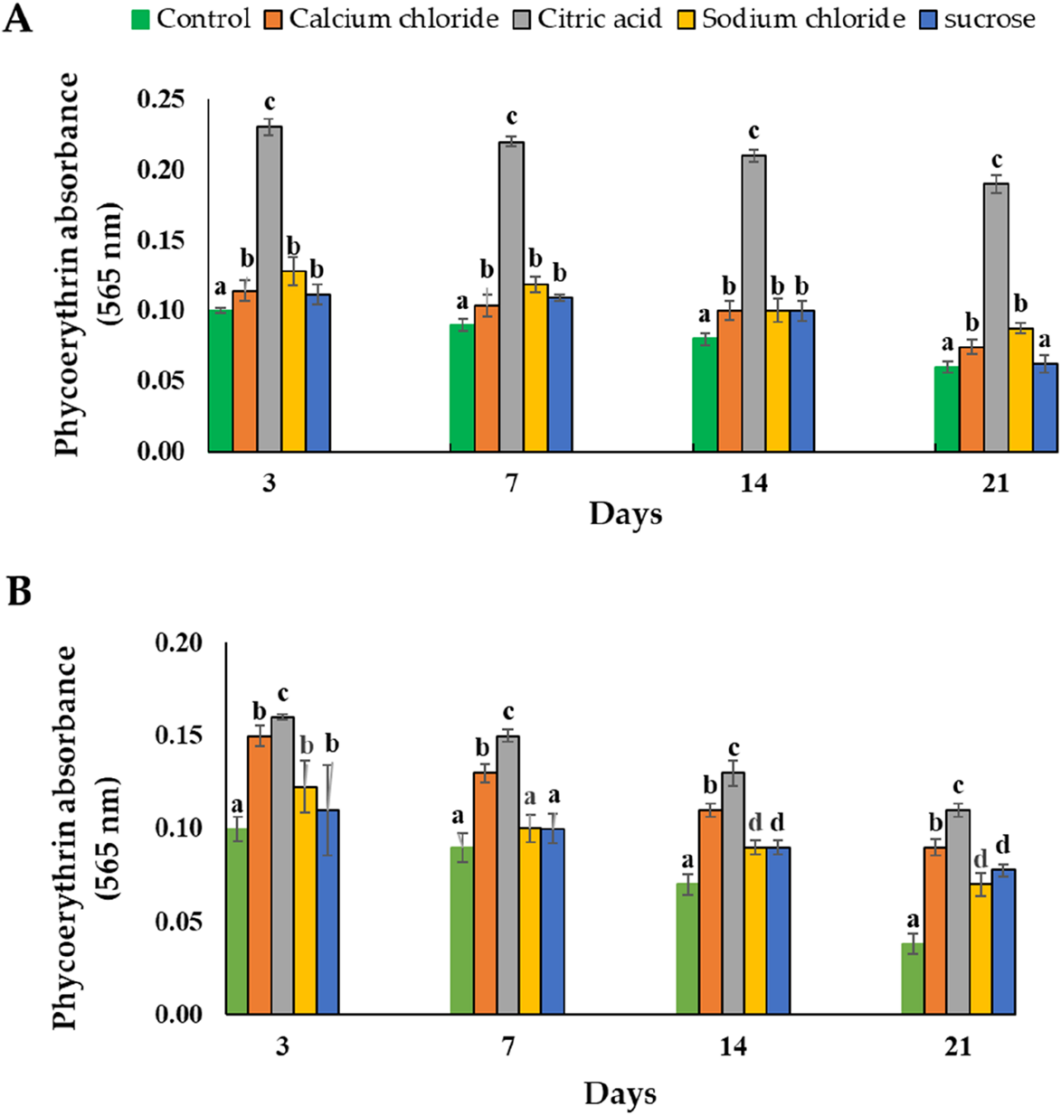
Stability of the purified C-PE in four stabilizer agents (calcium chloride, sodium chloride, citric acid, and sucrose) compared to control (water) in different storage times (3 to 21 days) at 4 °C (**A**) and 8 °C (**B**). The data were expressed as means ± SE of three independent experiments in triplicate each. The letters refer to comparisons within each day and treatment. The same letter indicates that data are not considerably different from one another (P < 0.05, ANOVA followed by Tukey’s grouping tests).

Based on absorption spectra, PE was classified into three groups: (i) B-PE, ג max ~ 540–560 nm, shoulder at ~ 495 nm (ii) R-PE, ג max ~ 565, 545 and 495 nm, (iii) C-PE, ג max ~ 565/543 and 492 nm ^29^. Purified PE absorption spectra displayed a maximum absorption at 562.8 nm (Fig. 2, Supp S1), which distinctly specifies that it can be classified as C-PE nature.

Data showed that the four additives, particularly citric acid, improved the C-PE stability remarkably when it was stored for 14 days at 4 °C in comparison with the control (Fig. 2A). However, during a long period (21 days) of storage at 4 °C, only calcium chloride, citric acid, and sodium chloridesignificantly improved C-PE stability, where citric acid was the more stabilizing agent. In addition, at high temperature (8 °C), the four stabilizing agents significantly improved C-PE stability for all storage times compared to the control, always with citiric acid as the most stabilizing agent, but the effect is less marked than at 4 °C (Fig. 2B).

**Figure 3:**
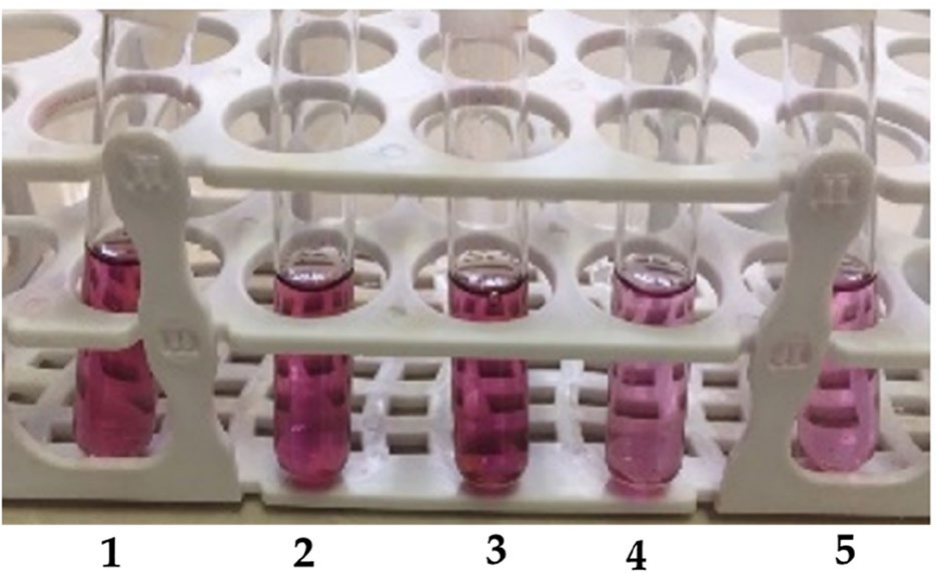
Color stability of the purified analytical grade C-PE in different preservative solutions: (**1**) citric acid, (**2**) calcium chloride, (**3**) sucrose, (**4**) sodium chloride, and (**5**) water (control) after storing for 7 days at 4 °C.

To conclude, the purified C-PE at a concentration of 565 µg/mL was more stable in citric acid solution (4 mg/mL) at both temperatures of 4 and 8 °C after it was stored for 21 days. Moreover, after 7 days of storage at 4 °C, the C-PE retained its color when dissolved in citric acid (Fig. 3). However, when the C-PE solution did not have t any preservative i.e., control, it showed discoloration after 7 days and completely discolored at 8 °C, but there was e no discoloration in the C-PE solution at 4 °C even after 21 days when stored in preservatives and particularely in citric acid.

### Evaluating MIC of the purified C-PE

The MICs of the purified C-PE, prepared in the citric acid as a stabilizer agent (CA+) or in water (CA−), against *E. coli* ATCC No.25922, and *Staphylococcus aureus* PTCC No. 1112 were presented in Table 2. The outcomes showed that the MIC of the purified C-PE in solution in citric acid was 3.9 µg mL^−1^ against the two bacterial model *E. coli* ATCC No. 25922 CA+ and *Staphylococcus aureus* PTCC No. 1112 CA+ . However, the MIC was two times higher (7.81 µg mL^−1^) against the two bacterial model in water solution (CA−) (Table 2). This shows that adding citric acid as a stabilzer agent enhances the stabitiy of the C-PE and, therefore, its antibacterial potential. Consequently, the purified C-PE at 3.9 µg/mL, corresponding to the MIC value, prepared in a citric acid solution (4 mg/mL) was further used to evaluate its antimicrobial and antioxidant potentials to increase the shelf life of fresh rainbow trout (*Oncorhynchus mykiss*) fillets.

**Table 2:** Minimum inhibitory concentration (MIC) in μg mL^−1^ against different bacteria strains of the analytical grade C-PE solubilized in citric acid (CA +) or in water (CA-). (-) no growth observed; ( +) growth observed.

Microorganism	565	250	125	62.5	31.25	15.62	7.81	3.9	1.95	0.97	MIC
*E. coli* ATCC No.25922 CA +	–	–	–	–	–	–	–	–	+	+	3.9
*E. coli* ATCC No.25922 CA–	–	–	–	–	–	–	–	+	+	+	7.81
*Staphylococcus aureus* PTCC No.1112 CA +	–	–	–	–	–	–	–	–	+	+	3.9
*Staphylococcus aureus* PTCC No.1112 CA-	–	–	–	–	–	–	–-	+	+	+	7.81

### Microbiological analysis

#### Total viable microorganisms and psychrophilic bacteria

**Figure 4:**
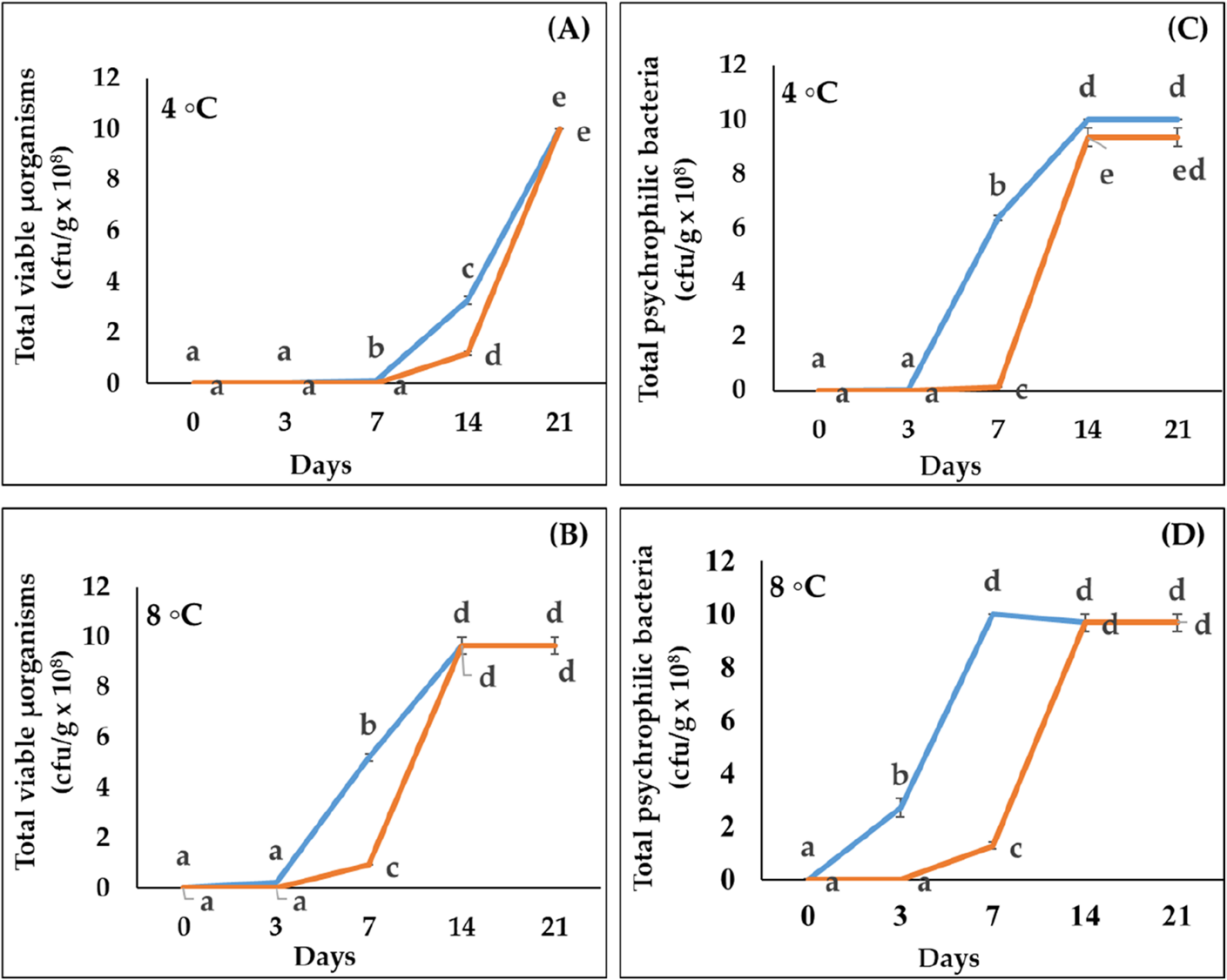
Total viable microorganisms count (cfu/g) of the treated rainbow trout fillets (orange curve) and control (blue curve) at 4 °C (**A**), and 8 °C (**B**) and total psychrophilic bacteria counts of the treated rainbow trout fillets (orange curve) and control (blue curve) at 4 °C (**C**), and 8 °C (**D**). The data were expressed as means ± SE of three independent experiments in triplicate each. The same letter indicates that the data are not very unlike each other (P < 0.05, ANOVA followed by Tukey’s grouping tests).

The results of the microbiological analysis, such as total viable microorganisms and psychrophilic bacteria, in trout fillet samples marinated with or without a solution of the purified C-PE (3.9 µg/mL) and stored for (0, 3, 7, 14 and 21) days at 4 and 8 °C which were presented in the Fig. 4. As shown in Fig. 4A, total viable microorganism counts in trout fillets, marinated in acitric acid soultion without C-PE (Control), increased significantly from 14 days of storage at 4 °C to reach a maximum on day 21. The curve of total viable microorganisms in fillet samples marinated in the C-PE (Fig. 4A) followed the same pattern as the control curve except that the increase in the count of these microoganisms was far less important on the 7 and 14 days. However, on day 21 the total viable microorganism counts reached the value of the control. At intenser heat (8 °C), the significant decline in the count of total viable microorganisms in treated fillets was observed only on day 7 in comparison with the control (Fig. 4B).

For psychrophilic bacteria, a similar effect than total viable microorganisms was observed at 4 °C, with a significant decrease of psychrophilic bacteria counts in treated trout fillets on days 7 and 14 in comparison with the the control (Fig. 4C). However, at 8 °C this difference was observedearlier on day 3 and up to only 7 days of storage (Fig. 4D, Supp S2).

In conclusion, the presence of C-PE at 3.9 µg/mL induced a significant reduction in overall viable microorganisms, and psychrophilic bacteria counts in the trout fillets at 4 °C.up to 14 days storing However, at 8 °C this reduction remained significant in only up to 7 days of storage.

#### Staphylococcal coagulase-positive bacteria and total coliforms

**Figure 5:**
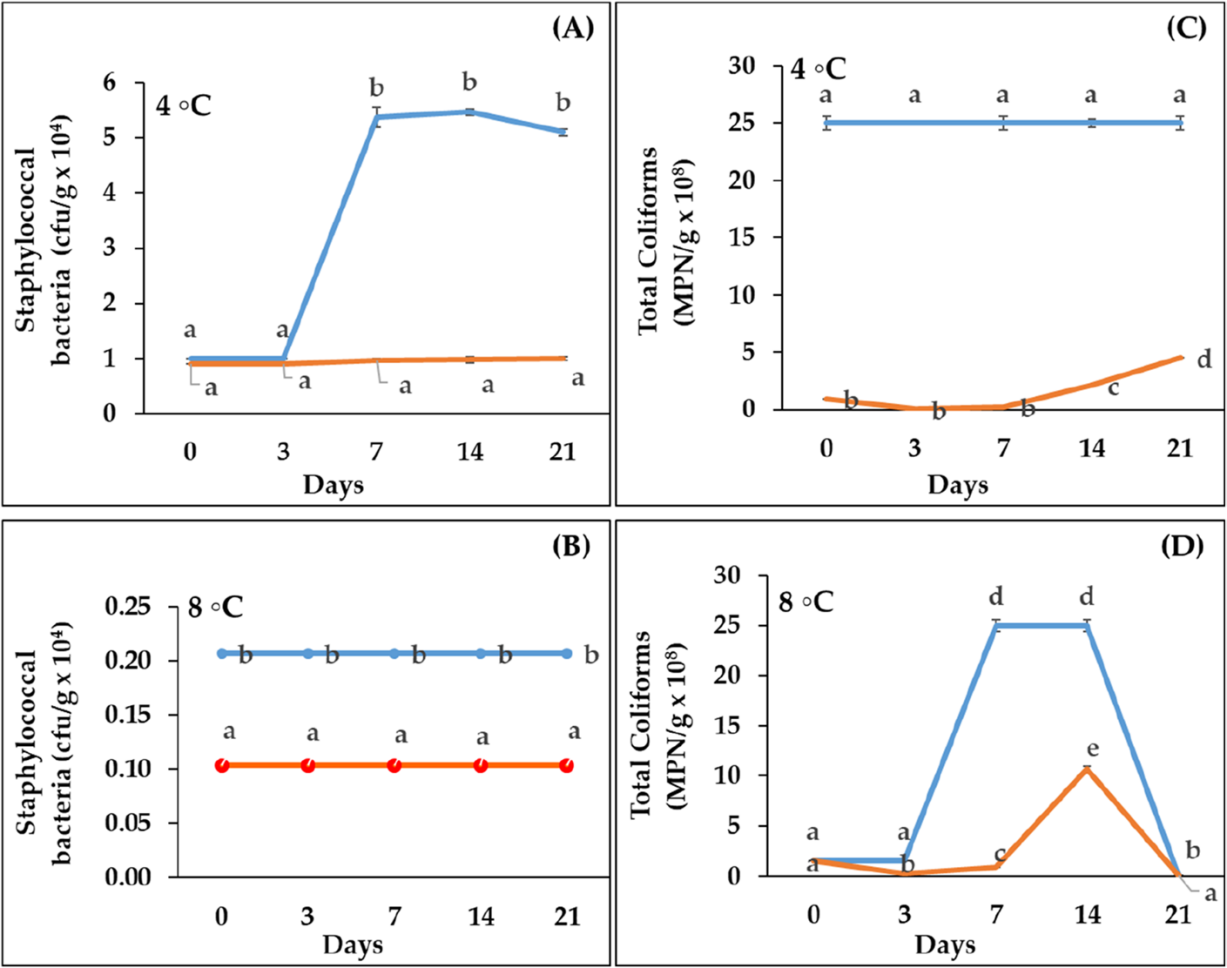
Staphylococcal coagulase-positive bacteria counts of the treated rainbow trout fillets (orange curve) and control (blue curve) at 4 °C (**A**), and 8 °C (**B**) and total coliforms of the treated rainbow trout fillets (orange curve) and control (blue curve) at 4 °C (**C**), 8 °C (**D**). The data were expressed as means ± SE of three independent experiments in triplicate each. The same letter indicates that data are not remarkably different from one another (P < 0.05, ANOVA followed by Tukey’s grouping tests).

The outcomes of C-PE impact at 3.9 µg/mL on Staphylococcal coagulase-positive bacteria and total coliform counts in trout fillets stored up to 21 days at 4 and 8 °C were presented in Fig. 5. As shown in Fig. 5A, the Staphylococcal coagulase-positive bacteria counts in untreated trout fillets (control) increased significantly until reaching a plateau after 7 days of incubation at 4 °C. However, in treated fillets, the count of bacteria remained stable at the level of the starting value and significantly considerably lower than the control throughout the entire period of storage (up to 21 days) at 4 °C. Although at 8 °C, the Staphylococcal coagulase-positive bacteria counts remained stable in treated and untreated trout fillets throughout the storage period, it was generally much lower in treated fish (Fig. 4B).

As for total coliforms, their level in untreated trout fillets remained stable but very high during the period of storage at 4 °C (Fig. 5C). Nevertheless, in treated fillets a slightly significant increase was observed after 14 days of storage, but the level always remained remarkably lower than the control throughout the entire period of storage (Fig. 5C). At 8 °C, the level of total coliforms in the control increased significantly after 7 days of storage and then dropped significantly until it reached its initial level in 21 days (Fig. 5D). In the treated trout fillets, the same pattern of the curve was observed, except that the level of total coliforms always remained significantly lower compared to the control.

In conclusion, the presence of C-PE at 3.9 µg/mL induced a significant reduction of Staphylococcal coagulase-positive bacteria and total coliform levels in the trout fillets up to 21 days of storage at 4 °C. However, when the temperature reached to 8 °C the above mentioned reduction remained significant up to21 days of storage for Staphylococcal coagulase-positive bacteria, but only up to 14 days of storage for total coliforms.

The presence of *E. coli* in the treated fillets and control during all periods of storage was confirmed by biochemical test (positive indole reaction). The *E. coli* found in the treated fillets throughout storage periods at 4 and 8 °C was less than the control, with zero *E. coli* in treated fillets at 4 °C on days 14 and 21 was recorded compared to *E. coli* in the control. Moreover, the presence of *Salmonella* spp. in the treated fillets and control was confirmed by biochemical test (negative urease reaction, negative indole test, TSI Test and H_2_S production). The presence of *Salmonella* spp. was detected at 4 °C in the control only on day 21 of storage. However, they were not found in treated fillets at the same temperature during the period of storage. At 8 °C, the presence of *Salmonella* spp. in the control was detected during the whole storage period, however, it was only found on days 0, 3 and 7 in treated trout fillets. The results of the serological analysis on the basis of polyvalent antisera showed that the species of *Salmonella* spp. detected belonged to groups of R, S, T, U, P and Q.

### The purified C-PE antioxidant activity

**Figure 6:**
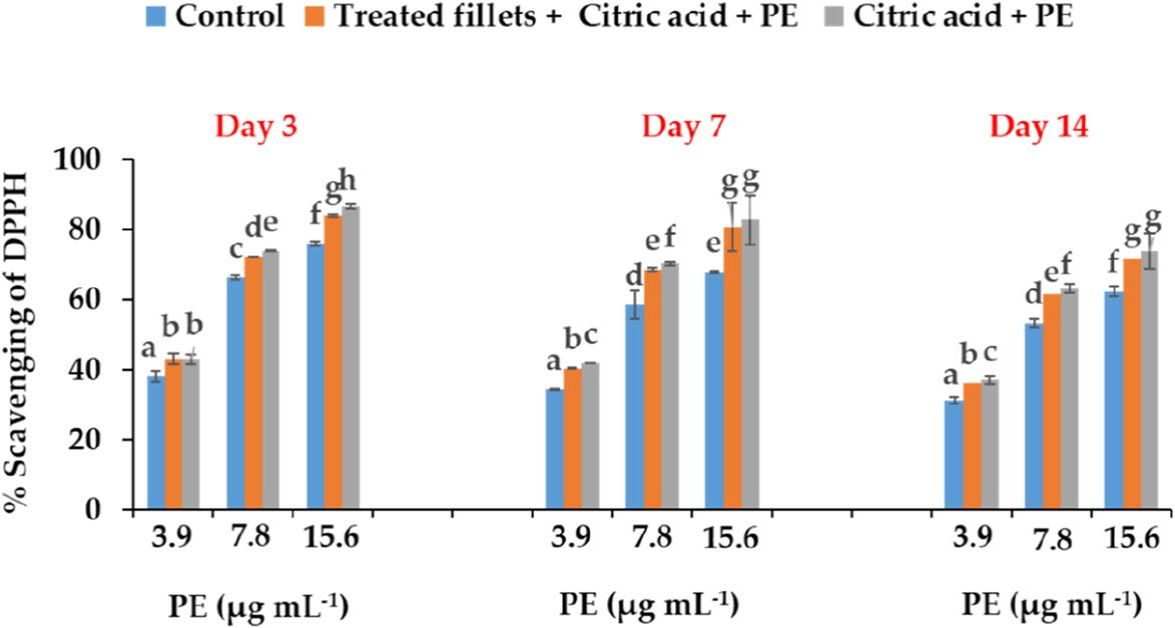
Free radical scavenging potential of analytical grade C-PE at different concentrations (3.9, 7.8, and 15.6 µg/mL) in water (control) and in citric acid (citric acid + C-PE), and of treated rainbow trout fillets with C-PE in citric acid (Treated fillets + Citric acid + C-PE) after 3, 7 and 14 days of storage at four °C. The data were expressed as means ± SE of three independent experiments in triplicate each. The same letter indicates that the data are not remarkably different from one another (P < 0.05, ANOVA followed by Tukey’s grouping tests).

We assessed free radical scavenging potential of the purified C-PE at three concentrations (3.9, 7.8, and 15.6 µg/mL) by the DPPH radical scavenging activity method in treated fillets compared to control at 4 °C during 3, 7, and 14 days of storage. As shown in Fig. 6, the purified C-PE in solution in citric acid preserved its antioxidant potential for all concentrations tested by scavenging the DPPH to 80% up to 7 days of incubation. However, for a longer period (14 days) its antioxidant potential dropped slightly but remained around 70%. In the treated trout fillets with the C-PE, the DPPH radical scavenging activity increased significantly throughout the storage period in dose dependent manner compared to the control, and it remained significantly similar to that of the C-PE in solution in the citric acid (Fig. 6).

In conclusion, from the low concentration corresponding to the MIC (3.9 µg/mL), the C-PE significantly enhanced the antioxidant potential of the treated trout fillets compared to the control and up to 14 days of storage. This antioxidative potential increased significantly with the dose to reach a value of 70% at 15.6 µg/mL on day 14.

### Results of sensory evaluation

**Figure 7:**
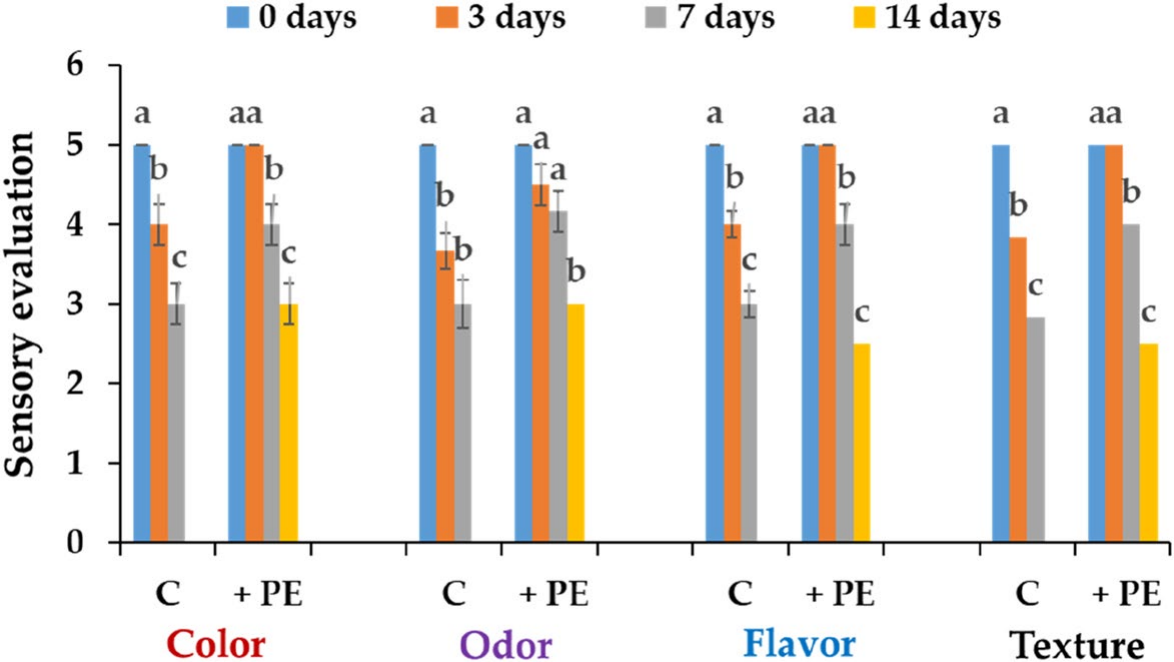
Sensory value of the rainbow trout fillets without (C) and with adding C-PE (+ PE) after 3, 7, and 14 days of storage at 4 °C. The data were expressed as means ± SE of three independent experiments in triplicate each. The same letter indicates that the data are not remarkably different from one another (P < 0.05, ANOVA followed by Tukey’s grouping tests).

Sensory values (color, odor, flavor, and texture) of tout fillets stored at different periods with C-PE at 3.9 µg/mL (up to 14 days) and in the control without C-PE (up to 7 days) were presented in Fig. 7. In the absence of C-PE, the color, odor, flavor, and texture of trout fillets deccreased significantly from 3 days of storage to reach a score of 3 (acceptable) on day 7 (Fig. 7). However, for the treated trout fillets, all of these sensory values remained at a high score level of 5 (extremely good) during the first 3 days of storage, and they substantially decreased to the score of 3 (acceptable) only on day 14 (Fig. 7). Moreover, after 7 days of storage of trout fillets in a C-PE solution of 3.9 µg/mL, the panelists considered the odor, flavor, and texture of fillets to be good.

In conclusion, at low concentration (3.9 µg/mL), corresponding to the MIC, C-PE significantly preserved the sensory quality parameters (color, odor, flavor and texture) of treated trout fillets to a good value in comparison with the control up to 7 days of storage.

## Discussion

Fish is a major source of unsaturated fatty acids, and high-quality proteins for humans; yet, it is extremely vulnerable to both oxidation and microbiological deterioration. In order to protect the health of consumers, the International Commission on Microbiological Specifications for Foods established the maximal restriction of 7.0 log CFU/g for aerobic microorganisms in fish^43^. the Shelf life can be increased through freezing and cold storage, yet, the quality of the fish is not entirely preseved^44^. Moreover, coating and using different chemical preservatives for increasing the shelf life of fresh fish fillets have been investigated^7,42,45–47^. However, the excessive use of chemical preservatives can generate unfavorible results on human health as well as on the nutrition amount of food. Hence, there is an urgent need to search for new natural agents, which are are not harmful and which are active against several food-borne pathogens and, as a result, can help in the preservation of food without altering its quality.The PBPs (PE, PC, and APC), covering 60% of the total cellular proteins and around 20% of the whole dry mass of the cyanobacteria, are among the value-added bioactive products isolated from cyanobacteria, which have given promising results as an ecological approach for inhibiting bacterial contamination of foods and thus increasing their shelf life^48^. The commercial production of PBPs was chiefly limited to the cyanobacterial genus *Arthrospira* (Spirulina)^49^ ^50–57^. Recently, other cyanobacterial species belonging to the genus *Anabaena*^58^ ^59^, *Synechococcus*^60^, *Phormidium*^58^, *Lyngbya*^61^, and the marine red algae *Amphiroa anceps*^62^ and *Porphyridium cruentum*^63^ have been explored. Lately, Limrujiwat et al. (2022) reported that six isolated cyanobacteria, *Phormidesmis* sp. RK02, *Leptolyngbya* sp. SCOM01, *Leptolyngbya* sp. SWL01, *Leptolyngbya* sp. LKK14, *Nostoc* sp. SW02, and *Scytolyngbya* sp. LKK05, made phycobiliprotein content with more than 400 mg g protein^-1.^when the highest yield (31.9%) was secured from *Nostoc* sp. SW02, with the ratio of phycoerythrin: phycocyanin: allophycocyanin 3.4:1.9:1.0.

In this study, we purified the PE from a marine cyanobacterial strain *Nostoc* sp. Ftsalt (MG549320) at analytical grade (C-PE) and evaluated its antimicrobial and antioxidant activities for the first time to increase the shelf life of fresh rainbow trout (*Oncorhynchus mykiss*) fillets. However, analytical grade C-PE is extremely sensitive to temperature^64^ and, therefore, for its use as an antimicrobial in the food industry and to make the process commercially viable, there is an essential need for research about a suitable preservative to enhance its stability^65^. Therefore, to investigate thermal stability of PBPs purified from a cyanobacterial strain *Oscillatoria* sp. BTA-170, Sharma et al.^66^ several monosaccharides like lactose, glucose, and fructose, were tested. Their results showed that glucose was found to be the most essential supplement enhancing the half-life of PE from 5.57 to 15.77 h at 85 °C. In this study, four industrial stabilizing agents (citric acid, calcium chloride, sodium chloride, and sucrose) were investigated at a soultion of 4 mg/mL. Our results showed that among these stabilizers, citric acid (4 mg/ml) was found to be suitable for C-PE stability for 14 days at 4 °C. Adding citric acid as a stabilizing agent improved the antibacterial potential of C-PE, hence its MIC (3.9 µg/mL) against *Echerichia coli* ATCC n °25,922 and *Staphylococcus aureus* PTCC n °1112, was halved from the one found in water (7.81 µg/mL).

Therefore, analytical grade C-PE (3.9 μg/mL) prepared in citric acid (4 mg/mL) has been used as a functional ingredient to upgrade the shelf life of fresh trout fillets and promote the consumer health. To increase the shelf life, the main microorganisms accountable for the decomposition of fresh trout fillets kept at refrigerated temperatures must, therefore, be reduced. The ICMSF^43^ recommended that the flesh total viable microorganism’s count should not exceed 10^6^ cfu/g wet weight. Our results showed that the overall pratcial microorganism’s counts in the trout fillets marinated in a C-PE solution and stored at 4 °C and 8 °C were below this maximum acceptable limit, until days 14 and 21, however it was until days 14 and 7 for control fillets, respectively. In addition, the total psychrophilic bacteria counts at 4 °C and 8 °C were below the maximum acceptable limit, 10^7^ cfu/g^43^, up to day 14 for treated fillets, but it was only until day 7 for the control. Concerning staphylococcal coagulase-positive bacteria, their total counts in the control fillets at 4 °C were below the maximum acceptable limit, 10^3^ cfu/g^43^ ^67^, up to day 7, however, for treated fillets, it was until day 14.

The limited number of the total coliforms can also be useful to specify the efficcay of safety measures throughout handling and of food^41,67^. In this analysis, the whole coliforms count in treated fillets span from 0.2 to 5 MPN/g at 4 °C, and from 0.93 to 11 MPN/g at 8 °C. However, in control fillets it ranged from 24 to 25 MPN/g at 4 °C and from 0.2 to 25 MPN/g at 8 °C. Moreover, C-PE also reduced the fecal coliforms count significantly at both temperature from 5 MPN/g in control fillets to 0–1 MPN/g in treated fillets. Similary, C-PE decreased the count of the more precise index of fecal contamination, that is *Escherichia coli* remarkably with zero colony recorded in treated fillets at 4 °C on days 14 and 21, compared to 5 colonies in the control. The existence of *E. coli* in foods means that the latter is contaminated with pollution of faecal origin and that it may, therefore, contain pathogenic microorganisms such as *Salmonella* spp. 0 cfu/25 g is the utmost microbiological limitation for the *Salmonella* count is that dissociates the products with good quality from those which have bad quality.^43^. Our results revealed that the *Salmonella* count of the control fillets at 4 °C were below the maximum acceptable limit, until day 14, however, it was until day 21 for treated fillets. Nevertheless, at 8 °C *Salmonella* spp. were detected both in control and treated fillets. The presence of *Salmonella* spp. found in fillets treated with C-PE after storage for 7 days at 8 °C may be due to contamination during the various stages of fish processing such as transport and handling or when it is washed or iced. In conclusion, the results obtained in this work clearly showed that the C-PE purified from a marine cyanobacterial strain *Nostoc* sp. Ftsalt (MG549320) significantly inhibits the increase of the count of most bacteria in harge of spoiling fresh trout fillets and thus prolongs their shef life in the refrigerator for at least 7 days. This is congruent with the earlier research, which have shown the antibacterial scope of cyanobacterial PE against various bacterial species. Indeed, Afreen and Fatma^29^ reported that PE exhibited antibacterial activity against Gram-negative as well as Gram-positive bacteria, with the strongest pre-emptive activity observed at 0.1 mg/mL against *Pseudomonas aeruginosa* MTCC2543, *E. coli* 25,922, and *S. aureus* MTCC902*.* Similarly, Sarada et al.^68^ manifested that PC from *Spirulina platensis* showed excessive antibacterial activity against *Klebsiella pneumoniae*, *E. coli*, *S. aureus*., and *P. aeruginosa.* Recently, Nowruzi et al.,^18^ demonstrated that PE exhibited also a strong inhibitory activity against* Bacillus subtilis,* and* Candida albicans.*

Bacterial contamination is one of the main resons that the fish and fish-products quality drops during storage, but it is not the only cause. Given their high content in polyunsaturated (26%) and monounsaturated (50%), fatty acids^69^, fish and fish-products are also very sensitive to lipid peroxidation during storage, which leads to a degradation in quality and the formation of unwanted odors. Many studies have been conducted that describe the extraction and purification of PE and PC from various algal and cyanobacterial species, the evaluation of their physicochemical properties^30,70–77^ their application as colorant in foods^34,78–80^, and their antioxidant potentiality but they were not added to the foods^81–83^. Nevertheless, as far as we are concerned, studies reporting the antioxidant activity of C-PE in food matrices to expent their shelf life are scarce. The antioxidant activity of PE purified from a marine cyanobacterial species *Halomicronema* sp. R31DM, was evaluated by three in vitro assyas namely, DPPH-radical scavenging activity, reducing power assay, and ferric-ion reducing ability of plasma assay^19^. The outcomes of the research revealed that PE exhibited substantial antioxidant activity probed by these 3 tests and the DPPH-scavenging activity of PE at 100 µg was considered to be similar to the positive control ascobic acid (80 µg), manifesting 100% scavenging. The protein sequences of the and-subunits of PE has 33 and 37 charge residues, respectively that have a capacity for sequestration of free radicals and metal ions and also the presence of linear tetrapyrrols in their structure. They are rich in doubles bonds and have the reducing ability, and both contribute to the total antioxidant activity of PE^19^. Our results showed, that for the first time the C-PE significantly enhanced the antioxidant potential in dose dependent manner in the treated trout fillets in comparison with the control, and up to 14 days of storage. The outcomes are congruent with other analyses that demonstrated that PE exhibeted greater free radical scavenging activity (IC_50_ = 0.023 mg/mL) than DPPH (IC_50_ = 0.043 mg/mL), and Superoxide anion radical (SOR) assay (IC_50_ = 0.553 mg/mL) ^29^. El Baky et al. ^84^ reported that the antioxidant potential of *Spirulina* is close (75.3% of DPPH radical scavenging activity) to that of the reference chemical antioxidant BHT (95% of DPPH radical scavenging activity). Similary, Shalaby andShanab^85^ noted that extracts of *Spirulina platensis* at 200 mg/mL exhibeted a DPPH radical scavenging activity up to 95.3%. These results have been confirmed and refined using encapsulated *Spirulina platensis* and a C-PC extract in dried noodles (1.5%, w/w) as an antioxidant in yogurts^86^, and cookies^84^, respectively. The results of these studies showed that yogurts supplemented with *Spirulina* and cookies supplemented with C-PC exhibited higher antioxidant activity throughout shelf life compared to products without the addition.

Moreover, human fish and fish-products eating is mainly reliant on the sensory attributes^87^, in which, color, odor, flavor, and texture are considered as the dominant factors for these foods quality. Because of their inherent bright color, easy availability, nontoxic proteinaceous nature, potential free radical scavenging ability, and water solubility, PBPs are extensively applied as a natural protein food coloring^88^. For example, the fillets of rainbow trout are highly sensitive to lipid oxidation throughout refrigerated storage, that cause the decrease in quality and formation of undesirable odor. During this study, we demonstrated that marinated fish fillets in C-PE significantly preserved their sensory parameters (color, odor, flavor and texture) to a good value compared to the control up to 7 days of storage. In addition, panelists preferred treated fish fillets with addition of C-PE until day 14 with acceptable mark. Hadiyanto et al.^51^ conversely, noted that dried noodles enriched by using phycocyanin extracted from *Spirulina* sp. at a high concentration (1.5%, w/w) were least favored by lecturers in term of colour, throughout sensory assay, yet showed the highest antioxidant capacity estimated by the DPPH method.

## Conclusion

Phycoerythrin with high purity (C-PE) was extracted from a marine cyanobacterial strain *Nostoc* sp. Ftsalt (MG549320). The addition of citric acid as a stabilizing agent improved its stability and antibacterial potential. At low concentration (3.9 µg/mL), this purified C-PE improved the microbiological quality of fresh rainbow trout fillets refregired at (4 °C), by significantly reducing the load of the main bacteria responsible for the spoilage on these fillets and extending their shelf life to at least 14 days. In addition, the purified C-PE remarkably enhanced the antioxidant potential in dose dependent manner in the treated trout fillets in comparison with the control, and up to 14 days of storage. The use of C-PE increased the antioxidant and microbiological shelf life of refrigerated fresh rainbow trout fillets and amplifies the sensory adequacy of these products when compared with those nonmarinated. Therefore, increasing the shelf life of fresh rainbow trout fillets would make it possible for trout producers in Iran to increase the length of time in the supply chain and make it more feasible for processors to maintain fresh, never-frozen rainbow trout fillets which can be sold in the local retail market or be exported.

### Supplementary Information

Supplementary Information 1.

Supplementary Information 2.

## Data Availability

The datasets used and/or analysed included in this published article [As supplemtray files, Supp S1, Supp S2].
